# High‐Resolution Elemental Analysis of Neuromelanin‐Containing Organelles in Human Locus Coeruleus

**DOI:** 10.1111/jnc.70531

**Published:** 2026-07-27

**Authors:** Zahraa Berro, Fabio A. Zucca, Maria Angels Subirana, Adrian‐Marie Philippe, Dirk Schaumlöffel, Anaïs Carpentier, Andrea Capucciati, David Bouvier, Michel Mittelbronn, Jean‐Nicolas Audinot, Luigi Zecca, Antje Biesemeier

**Affiliations:** ^1^ Advanced Instrumentation for Nano‐Analytics, Scientific Instrumentation and Process Technology Luxembourg Institute of Science and Technology (LIST) Belvaux Luxembourg; ^2^ Faculty of Science, Technology and Medicine (FSTM) University of Luxembourg Esch‐sur‐Alzette Luxembourg; ^3^ Institute of Biomedical Technologies National Research Council of Italy Segrate Milan Italy; ^4^ Institut des Sciences Analytiques et de Physico‐Chimie Pour l'Environnement et les Matériaux (IPREM), UMR 5254, CNRS Université de Pau et des Pays de l'Adour Pau France; ^5^ Advanced Characterization of Surface, Interface and Structure, Advanced Analysis and Support Luxembourg Institute of Science and Technology Belvaux Luxembourg; ^6^ National Center of Pathology Laboratoire National de Santé (LNS) Dudelange Luxembourg; ^7^ Department of Chemistry University of Pavia Pavia Italy; ^8^ Pezzoli Foundation for Parkinson's Disease Milan Italy; ^9^ Luxembourg Centre of Systems Biomedicine (LCSB) Esch‐sur‐Alzette Luxembourg; ^10^ Department of Health, Medicine and Life Sciences (DHML) University of Luxembourg Esch‐sur‐Alzette Luxembourg; ^11^ Division of Neuropathology, Department of Pathology and Neuropathology, Medical Faculty University of Cologne Cologne Germany

**Keywords:** brain aging, EDX, EM, locus coeruleus, nano‐SIMS, neurochemical analysis, neuromelanin

## Abstract

Neuromelanin‐(NM) containing organelles are sub‐cellular auto‐lysosomal structures composed of three main compartments: NM pigment, protein matrix, and lipid bodies. These organelles accumulate during aging and are found predominantly in the catecholaminergic neurons of Substantia Nigra (SN) and Locus Coeruleus (LC), the main regions affected in Parkinson's disease (PD). NM serves a protective function by sequestering potentially toxic metals like Cu, Fe, and Al. However, NM released from degenerating neurons may lead to a cascade of events resulting in neuroinflammation and neurodegeneration. Therefore, elemental analysis of NM‐containing organelles presents a crucial step to understand aging and PD. In LC, such studies are limited because NM isolation requires large postmortem cohorts and analyses may be impaired by tissue processing. By integrating high resolution electron microscopy (EM), nano‐secondary ion mass spectrometry (nano‐SIMS), and energy dispersive X‐ray (EDX) microscpectroscopy, the elemental composition of intact NM‐containing organelles was analyzed in seven postmortem LC tissues. Chemical mapping with down to 5–10 nm lateral resolution (EDX) fostered discrimination of structural composition (N, P, S, Cl) and metal storage (Al, Ca, Fe) across neurons and within individual NM‐containing organelle sub‐compartments (with diameters down to 0.2 μm for lipid bodies) from the same sample. NM‐containing organelles were identified by an elemental fingerprint pattern. Metals accumulations were localized predominantly to the NM pigment compartment identified by its pheomelanin‐rich portion (S). This confirms NM's role in accumulating physiological as well as potentially toxic metal species. Moreover, semi‐quantitative analyses provided insights into inter‐ and intra‐subject NM metal accumulation, showing that S and Fe exhibited a positive aging trend. Hemispheric asymmetry was observed for Al, Ca, and Fe, with higher levels observed in NMs of the right brain hemisphere suggesting region‐specific accumulation that warrants further investigation to better understand aging‐related changes and the neuronal vulnerability of the LC in PD.

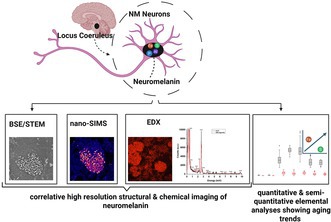

## Graphical abstract

Created in https://BioRender.com. Neuromelanin (NM), a dark metal‐chelating pigment in the brain, has long been of interest for its potential roles in aging and Parkinson's disease. In this study, we explored NM‐containing neurons (NM neurons) in the human locus coeruleus at unprecedented resolution. Using correlative electron microscopy (BSE/STEM), nano‐secondary ion mass spectrometry (nano‐SIMS), and energy‐dispersive X‐ray spectroscopy (EDX), we identified and mapped the distribution of metals (Al, Ca, Fe) and structural elements (N, P, S, Cl) contained within the NM‐containing organelles and their sub‐organellar compartments. In addition, quantitative, and semi‐quantitative analyses revealed positive trends with aging for S and Fe, highlighting the potential of these approaches for future studies involving larger aging cohorts or Parkinson's disease samples.

**Abbreviations**
AD: Alzheimer's disease
at%: atomic percent
BSE: backscattered electron imaging
DF‐LM: dark‐field light microscopy
EDX: energy dispersive X‐ray microanalysis
EELS: electron energy loss spectroscopy
EPR: electron paramagnetic resonance
LAMMA: laser microprobe mass analyzer
LC: locus coeruleus
MRI: magnetic resonance imaging
Nano‐SIMS: nano‐secondary ion mass spectrometry
NM: neuromelanin
PD: Parkinson's disease
PIXE: particle‐induced X‐ray emission
SN: substantia nigra
STEM: scanning transmission electron microscopy
STXM: synchrotron soft X‐ray spectro‐microscopy
TEM: transmission electron microscopy

## 1 Introduction

Neuromelanin (NM) is a black‐brown pigment typically localized within the dopaminergic and noradrenergic neurons of the substantia nigra (SN) and locus coeruleus (LC) (Zecca et al. 2004), two brain areas that are selectively affected in Parkinson's disease (PD) and Alzheimer's disease (AD) (Zarow et al. 2003). Smaller amounts of NM were also found in other regions of the human brain (Zecca, Bellei, et al. 2008). Electron microscopy imaging of the SN and other pigmented brain regions showed that NM‐containing organelles exhibit a distinct structure comprising three compartments with different electron density features: an electron‐dense portion composed of the NM pigment, an intermediate electron‐dense compartment likely constituted by a protein matrix and an electron‐transparent lipid compartment represented by lipid bodies (Duffy and Tennyson 1965; Sulzer et al. 2008; Zecca, Bellei, et al. 2008).

NM starts to accumulate in catecholaminergic neurons of both the SN and LC early in life and continues to accumulate linearly until the ninth decade (Zecca et al. 2004). Despite being abundant in healthy aged subjects, NM levels are heavily reduced in the SN of individuals with PD (Zecca et al. 2002). This reduction is even visible with the naked eye in postmortem SN tissues of PD patients, as first reported by Konstantin Nikolaevich Tretiakoff in his doctoral thesis in 1919 and attributable to the loss of pigmented neurons (Lees et al. 2008). A recent NM‐sensitive magnetic resonance imaging (MRI) study reported that the loss of NM in PD patients was even more prominent in the LC as compared to the SN (Liu et al. 2023), which was in line with the observation of a greater neuronal loss in LC postmortem tissue (Zarow et al. 2003). Therefore, the loss of NM‐containing SN and LC catecholamine neurons is considered an important factor contributing to the subsequent clinical motor and non‐motor symptoms of PD (Kouli et al. 2018).

NM of the SN can chelate and accumulate iron, with higher iron accumulation observed in PD patients, as demonstrated in an early energy dispersive X‐ray (EDX) analysis by Jellinger and colleagues (Jellinger et al. 1992). Consistently, NM isolated from both LC and SN of healthy aged subjects and analyzed by electron paramagnetic resonance (EPR) spectroscopy in later works reflected the accumulation and sequestration of iron by NM in both regions (Zecca et al. 2004), as well as other pigmented regions of the human brain (Zecca, Bellei, et al. 2008). The ability of NM to sequestrate iron, but also other metal ions like copper and zinc, together with other toxic metals including aluminum, lead, and mercury in different brain regions, serves a protective function for the neurons (Zecca, Bellei, et al. 2008). This is confirmed by the ability of NM to protect against iron‐mediated oxidative processes (Zecca, Casella, et al. 2008). On the contrary, NM can exert toxic effects: when released by dying neurons, NM activates microglia and induces inflammation, resulting in further degeneration of dopaminergic neurons in the SN, contributing to the progression of neurodegenerative mechanisms (Wilms et al. 2003; Zhang et al. 2011).

NM has two different iron binding sites, a high‐affinity and a low‐affinity binding site (Double et al. 2003; Zucca et al. 2023). When iron is being sequestered by the high‐affinity binding site, NM exerts a protective role (Zecca, Casella, et al. 2008). In certain conditions, when the iron concentration in the brain is increased, the high‐affinity binding sites of NM become saturated, which leads to iron binding to low‐affinity binding sites. Here, iron can be easily released and may promote redox reactions, including Fenton's reaction, thus leading to neurotoxicity (Zecca, Casella, et al. 2008; Zucca et al. 2023).

Although postmortem studies sometimes make it difficult to establish cause and effect, substantial chemical alterations of intraneuronal NM in the LC and SN may contribute to different neuronal vulnerability (Filimontseva et al. 2025). Differences in NM composition between the LC and SN, including variations in metal binding and structural components (Wakamatsu et al. 2015; Zecca et al. 2004), may underlie region‐specific susceptibility to NM‐related stress contributing to the earlier degeneration of LC neurons observed in PD (Iannitelli and Weinshenker 2023). Thus, analysis of NM in catecholaminergic neurons is crucial for understanding its role in neurodegeneration.

The low solubility and limited availability of NM pigments, particularly those isolated from small human brain regions such as the LC, make their analysis by conventional analytical methods a hard task. Indeed, obtaining a yield of 1 mg of NM requires 4–5 human SN samples, whereas more than 30 human LC samples are needed to obtain < 1 mg of NM (Engelen et al. 2012). Therefore, it would be highly advantageous to analyze NM's chemical composition and ultrastructure directly within preserved brain tissue using high‐resolution chemical imaging techniques. In 1992, Good and colleagues used laser microprobe mass analysis (LAMMA) to examine iron and aluminum accumulation in NM‐containing neurons of the SN in both PD and control subjects. Their findings revealed a higher iron accumulation within NM granules of the PD patients (Good et al. 1992). Recent studies used synchrotron soft X‐ray spectro‐microscopy (STXM) to characterize iron deposition in NM‐containing cells of the SN at high spatial resolution, further confirming that NM clusters are laden with iron (Brooks et al. 2020). Moreover, particle‐induced X‐ray emission (PIXE) elemental mapping, performed with a sub‐500 nm lateral resolution, demonstrated that iron is confined to NM‐containing organelles in the SN; however, without concentration difference between the PD and a control case (Reinert et al. 2007). More recently, and using a larger sample size, a similar approach has demonstrated a higher iron load of NM in PD subjects compared to controls (Friedrich et al. 2021). In addition, the analysis of NM‐containing organelles in the SN of healthy aged individuals by correlative energy‐dispersive X, ‐ray spectroscopy (EDX) and nano‐secondary ion mass spectrometry (nano‐SIMS) showed iron accumulation and colocalization within the NM pigment of NM‐containing organelles with even below 150 nm lateral resolution (Biesemeier et al. 2016). While the aforementioned studies focused mainly on the SN‐NM, high resolution chemical and elemental imaging of LC‐NM remains absent, highlighting an important gap in the existing literature.

In this study, high‐resolution elemental imaging techniques, namely nano‐SIMS and scanning transmission electron microscopy coupled with EDX mapping (STEM‐EDX) with lateral resolutions down to 5 nm for EDX, were implemented for investigating LC‐NM in human brain samples. This approach yielded unprecedented insight into the metal accumulation and distribution, mainly of iron, in intact NM‐containing organelles of the LC neurons. The use of a single brain tissue section per analyzed sample avoided the need for a large sample size and probable alterations due to the chemical changes occurring during the NM isolation procedure.

## 2 Materials and Methods

### 2.1 Ethics Statement

Ethical statement: This study was approved by the Research Ethics and Integrity Committee of the National Research Council of Italy (CNR), with Protocol N. 0092788/2022, and the Comité National d'Ethique de Recherche (CNER N° 202 104/01) in Luxembourg.

### 2.2 Brain Tissues

Seven LC tissue samples were obtained from four anonymized healthy subjects: for three subjects, both left and right LC were collected, while for one subject only left LC was available (see Table 1 for further information on the samples). The LC tissue samples were obtained during autopsies of subjects who died without evidence of neuropsychiatric and neurodegenerative disorders. Subjects included in this study did not show any neurological or vascular alterations at pathological examinations. Written informed consents for using brain samples for research purposes were obtained from closest relatives and are stored at the Institute of Legal Medicine and Insurances, Department of Biomedical Sciences for Health, University of Milan, Milan, Italy. All tissue samples were analyzed anonymously.

**TABLE 1:** List of samples with their ID reference and other information like brain hemisphere region, sex, and age of the subjects. The ID informs on subject number (1–4), left or right brain hemisphere (L or R), and the age (75–86 years).

Sample ID	Brain area	Sex	Age
S1L‐75	Locus Coeruleus‐left	Male	75
S2L‐79	Locus Coeruleus‐left	Male	79
S2R‐79	Locus Coeruleus‐right	Male	79
S3L‐86	Locus Coeruleus‐left	Female	86
S3R‐86	Locus Coeruleus‐right	Female	86
S4L‐78	Locus Coeruleus‐left	Male	78
S4R‐78	Locus Coeruleus‐right	Male	78

Tissues were fixed in 2% glutaraldehyde buffered in cacodylate (0.1 M, pH 7.4) immediately after autopsy, dehydrated in a graded series of ethanol, and embedded in epoxy resin (Epon) without further contrast enhancement by osmium or uranium salts during the embedding procedure.

Using a *LEICA ULTRACUT UCT ultramicrotome* with a 45° diamond knife from DIATOME, Switzerland, the tissues were sectioned at 100 nm thin sections for TEM‐EDX and placed on a standard 100 square mesh gold grid (21‐3GM100, Micro to Nano Innovative Microscopy Supplies; Netherlands) without formvar to reduce background signal. As they proved to be extremely fragile without support film, the grids were first imaged by 200 kV TEM to find areas of interest for the analysis, then removed from the TEM and coated from the backside with 10 nm carbon film using the ACE600 coater from Leica (Wetzlar, Germany) to enhance section stability under the electron beam without introducing additional elements.

For nano‐SIMS, the same tissue blocks were sectioned to 250 nm semi‐thin sections and placed on silicon wafers (SIEGERT WAFER, Aachen, Germany). BSE imaging was performed on the same region of interest (ROI) in an adjacent serial section after post contrasting of the tissues using uranyless and lead citrate (DELTA MICROSCOPIES, Mauressac, France) in order to enhance contrast.

For all the downstream analysis, several NM‐containing organelles per neuron were analyzed within each ultramicrotome section per sample. Only sections containing at least 3 pigmented neurons were selected.

### 2.3 Chemical Imaging by Energy Dispersive X‐Ray Spectroscopy (EDX)

A JEOL JEM F‐200 cold FEG microscope operating at an acceleration voltage of 200 kV and equipped with dual JEOL 100 mm^2^ Silicon Drift Detectors (Tokyo, Japan) was used for correlative high‐resolution ultrastructural analysis and EDX mapping. STEM‐EDX maps were acquired with 4.6 ms/pixel dwell time for an overall 512 × 512 pixel size and further analyzed using *JEOL Analysis Station software V.4*. For each pixel in a scanned area of interest, an EDX spectrum of counts versus energy (keV) was acquired, from which the distribution maps of elements of interest can be generated. Quantitative analysis is automatically obtained by means of instrument‐specific standard factors for each element and using the Cliff Lorimer equation (Egerton 2005).

For the comparison of significant elemental distributions in maps with the corresponding raw integrated spectra from the same ROI, note that although an element may be detectable in the integrated EDX spectrum of the entire mapped field of view, it may appear weak or even undetectable in the corresponding maps, as the counts are distributed across many pixels. This does not necessarily mean that an element is absent in a certain ROI if it is not detected in the map, but it might only be present in small amounts below the detection limit of the visualization method. To supervise and confirm the automatic peak detection and quantification of the analysis software, spectra were re‐evaluated manually and a peak was only considered significant when its intensity was three times higher than background noise. Otherwise, the atomic% (at%) value was manually set to “0” for that peak and the sum of signals recalculated to 100%. To account for any extrinsic factors such as differences in acquisition conditions, background contributions, or specimen thickness variations that vary from one measurement to another, EDX spectra were normalized to an internal reference peak, here Co‐Kα. The Co signal detected in the spectra cannot originate from the material itself and must reflect artifact contributions—such as instrumental background or spurious X‐ray signals—that are common to all acquisitions. Another point to consider that had to be manually supervised was the fact that Fe can be contained not only in the sample, but is, like Co, also a typical artifact peak coming from the column. To enable quantitative assessment of Fe storage in NM pigment in the NM‐containing organelles, first, three EDX maps from three different neurons were acquired per tissue section. Within each ROI (EDX map), several areas were selected that were either indicative of NM pigment or background signal (cytoplasm). Second, the NM pigment spectra were normalized to the cytoplasm spectra using a scaling factor calculated as the ratio of the integrated Co signal in the pigment to that in the cytoplasm. Finally, normalized spectra were partially smoothed using the Savitzky–Golay smoothing approach in *Origin* with a window size of five.

As quantitative at% values for Fe were close to the detection limit of 0.02 at% for all samples, a second approach was used to confirm quantitative measurements. The ratio of Fe to Co net intensities, the so‐called relative Fe signal, was calculated to obtain the pure sample‐based Fe content of the spectra without contribution of signal from the column.

Overlay images of spectral mappings were generated using the *Correlia* image registration plugin in *Fiji* (*ImageJ*, Rohde et al. 2020).

### 2.4 Nano‐Secondary Ion Mass Spectrometry Imaging and Data Treatment

Nano‐SIMS analysis was performed on a Cameca NanoSIMS50L (Gennevilliers, France), equipped with two primary ion sources allowing parallel multi‐mass detection of positive or negative ions. The Cesium (Cs^+^) source, with a primary current of 0.22 pA, was used for the detection of secondary negative ions: ^12^C_2_^−^ (m = 24.0 amu), ^12^C^14^N^−^ (m = 26.00307 amu), ^32^S^−^ (m = 31.97207 amu), and ^31^P^16^O_2_^−^ (m = 62.96359 amu). The Oxygen (O^−^) source was tuned to 10 pA for the analysis of positive ions, including ^12^C^+^ (m = 12.0 amu), ^27^Al^+^ (m = 26.98154 amu), ^40^Ca^+^ (m = 39.96259 amu), ^56^Fe^+^ (m = 55.93494 amu), ^63^Cu^+^ (m = 62.9296 amu), and ^66^Zn^+^ (m = 65.9260 amu). The carbon signal (^12^C_2_^−^ and ^12^C^+^), which mainly originates from the resin, was used to ensure signal homogeneity across the entire image and to normalize analyte signals to the major matrix element (carbon). The instrument was tuned to reach a Mass Resolution Power of M/ΔM < 7000. The detectors were mass calibrated prior to the measurements, using reference standard materials prepared in the laboratory on a silicon wafer substrate.

Maps with a field of view (FOV) of 40–45 μm were used to visualize the composition of the NM‐containing neurons down to their sub‐organellar compartmentalization. For each sample, three distinct NM‐containing neurons within the same slice were analyzed for their spatial elemental distribution. Mapping was performed with a 256 × 256 pixels matrix, with a scanning dwell time of 20 ms/pixel for both positive and negative ion detection. For each area, successive image planes were acquired and then summed into a single image to increase pixel‐level signal intensity. Specifically, 10 planes were integrated for positive mode and 3 planes for negative mode after a drift correction in *OpenMIMS* plugin in *ImageJ* software (USA) using the auto track option. This ensures accurate alignment of multiple planes and preserves high spatial resolution. The same software was then used to stack the subsequent planes into a single image. Spatial binning was then applied by a 2 × 2 binning factor, reducing the pixel resolution from 256 × 256 pixels to 128 × 128 pixels to further enhance the signal intensity. Finally, regions of interest were selected on each map. The counts per pixel values were calculated to determine the intensity per area for each ROI, and these values were then normalized to the carbon signal.

Overlay images were generated using the *Correlia* image registration plugin in *Fiji* (*ImageJ*, Rohde et al. 2020).

### 2.5 Back‐Scattered Electron (BSE) Microscopy for NM Verification

Sections of 250 nm thickness, consecutive to those analyzed by nano‐SIMS, were imaged on a Zeiss Sigma 300 SEM (Oberkochen, Germany) using a backscattered detector at an acceleration voltage of 20 kV and a beam current of 100 μA. Acquisition of the final 1536 × 2048 pixels BSE images was performed at a dwell time of 0.41 ms/pixel.

### 2.6 Statistics

Nano‐SIMS normalized counts/pixel ratios and EDX mean atomic percentages (at%) were calculated using Microsoft Excel 365 (Version 2603), with results expressed as mean ± standard deviation. For data presentation, a single distinct outlier (17.61) was excluded from the ^27^Al^+^ box plot of the S3R‐86 sample in Figure 3. Linear relationships were assessed using the two‐tailed Spearman rank correlation coefficient with a significance threshold set at *p* ≤ 0.05 in OriginPro 2019b (Version 9.6.5.169).

## 3 Results

### 3.1 Composition of NM and Lipid Bodies of NM‐Containing Organelles by Nano‐SIMS

#### 3.1.1 Identification of the NM‐Associated Compartments by Means of Correlative Microscopy and Microanalysis

**FIGURE 1:**
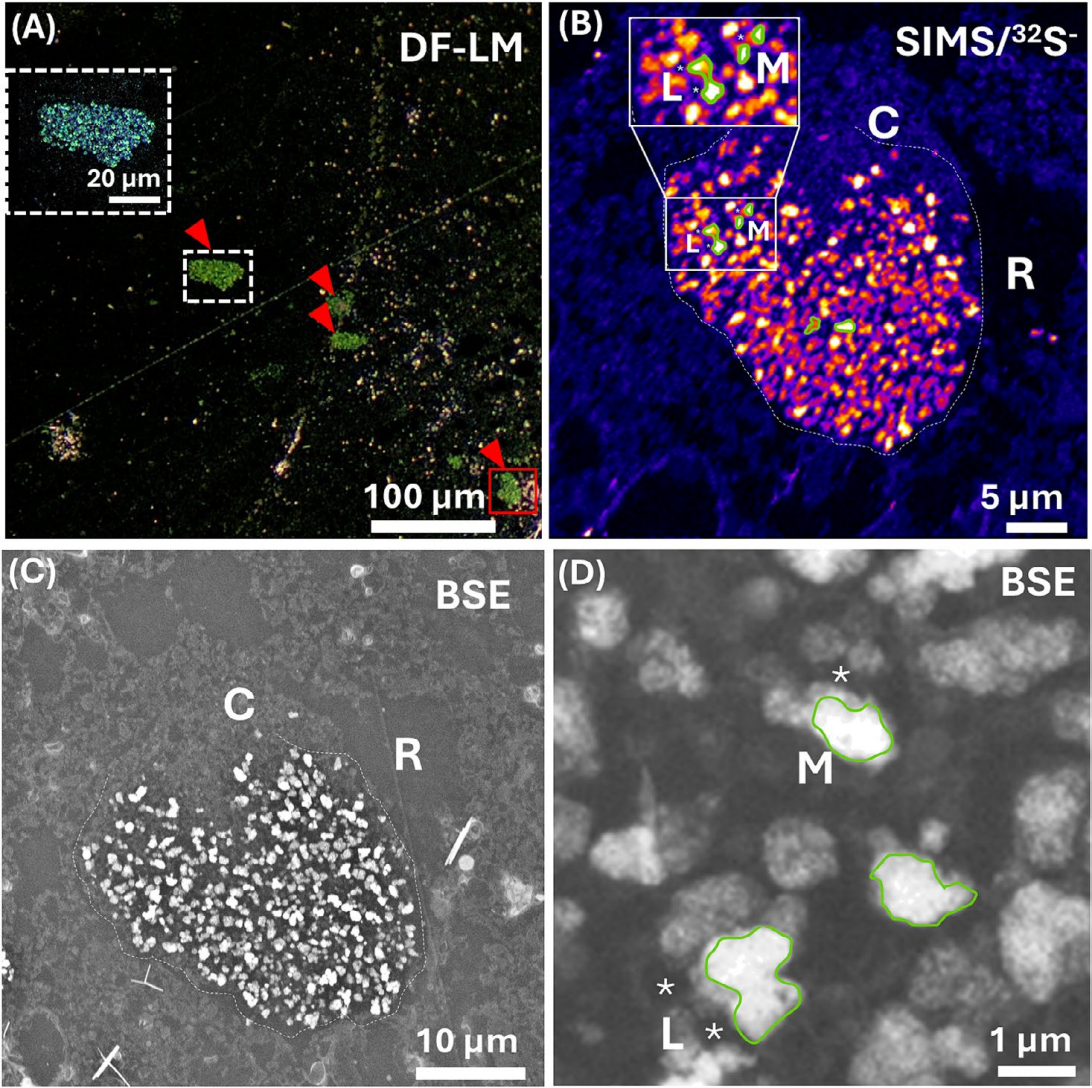
Representative correlative microscopic analyses of LC tissues showcasing the identification of noradrenergic pigmented neurons by their NM‐based auto contrast in LC tissues. (A) Low magnification dark field light microscopy (DF‐LM) image of the LC section. Red arrows indicate auto contrasting pigmented neurons; the red square marks an exemplary ROI selected for nano‐SIMS, and the dashed white squares mark another neuron at different magnifications (insert). (B) ^32^S^−^ sulfur map obtained from nano‐SIMS of the NM‐containing neuron selected in A. Sub‐regions are labeled as follows: C = cytoplasm, *R* = resin, M = NM pigment and L = lipid compartment of NM‐containing organelles. (C) Back‐scattered electron (BSE) image of the same neuron on a consecutive section. The electron‐dense NM pigment appears bright and dashed lines indicate the approximate cell boundaries. (D) A zoomed‐in view of the BSE image showing the ultrastructural compartments. Asterisks in B and D denote the roundish lipid bodies (L), while green outline lining indicates the NM pigment (M).

With correlative dark‐field light microscopy (Figure 1A), nano‐SIMS elemental mapping (Figure 1B) and electron microscopy (Figure 1C,D) relevant areas of interest were identified and further characterized. This work focused on pigmented noradrenergic neurons of the LC that were detected by means of their sizes (~15–40 μm, Giorgi et al. 2020) and the characteristic contrast of their NM‐containing organelles. In dark field light microscopy (DF‐LM), the NM‐containing organelles represent characteristic yellow‐green auto contrast (Figure 1A, red arrowheads indicate NM‐containing neurons), which originates from the intrinsic light‐scattering contrast of the NM structure (Lawana et al. 2020). The typical dark color of the NM pigment that is usually obtained by bright field light microscopy imaging was not clearly visible in ultrathin sections. This color, originating from NM pigment being composed of a mixture of optically black eumelanin and reddish pheomelanin moieties (Bush et al. 2006; Wakamatsu et al. 2003) can however be tracked by chemical imaging as follows. Pheomelanin represents about 20%–25% of the total NM pigment portion (Wakamatsu et al. 2003; Bush et al. 2006), and is composed of benzothiazine and benzothiazole groups formed by the incorporation of cysteine into dopamine derivatives (Bush et al. 2006; Crippa et al. 2010; Monzani et al. 2019; Wakamatsu et al. 2003, 2012). The contribution to S content by cysteine/cystine in proteins bound to NM is much lower than that of NM from benzothiazine and benzothiazole units (Engelen et al. 2012; Zecca et al. 2000). Consequently, pheomelanin portion is a sulfur‐rich structure, rendering sulfur a marker of the NM pigments in the elemental distribution analysis.

Nano‐SIMS (like EDX, presented later) is a technique which enables the acquisition of chemical maps revealing the spatial distribution of metallic and non‐metallic elements, like sulfur, thereby allowing the analysis of whole‐cell chemical composition at subcellular resolution with high sensitivity. Therefore, nano‐SIMS was used to highlight NM pigment in NM‐containing organelles by means of their highest sulfur signal intensity in ^32^S^−^ maps (Figure 1B, same neuron imaged as in Figure 1A) and later for metal storage analysis in the same maps. Particularly S‐rich areas with typical sizes between 0.5 and 2.5 μm in diameter were identified as the NM organelles, in which the NM pigment compartment showed the highest sulfur signal (as confirmed with BSE, see below). Structures showing minor or no S signal were considered as protein matrix and lipid bodies of NM‐containing organelles, respectively. In contrast, structures with dark blue color signals outside the NM‐containing organelles are other cytoplasmic non‐soluble materials with lesser S content (labeled C in Figure 1B). During the embedding process, resin infiltrates structures that were previously occupied by cytoplasmic water‐soluble materials: therefore, it occupies these spaces after the different preparation and washing steps. As a result, the resin exhibits very low electron density in electron microscopy (see below) and appears as black void areas lacking signal in the S maps (R, Figure 1B).

For further confirmation, post‐SIMS backscattered electron (BSE) imaging of the same selected NM‐containing neurons was performed on consecutive sections, revealing the typical morphology of pigmented neurons with multiple NM‐containing organelles in their cytoplasm (Figure 1C). NM pigments (M) and lipid bodies (L) of the NM‐containing organelles are depicted clearly in the zoomed BSE image, highlighting several NM‐containing organelles (Figure 1D). As compared to TEM images known from the literature (Zucca et al. 2018), the BSE images show an inverted color scheme, in which the electron dense structures (the melanic portion of NM pigments) appear white with a size range of 0.5–1.5 μm in diameter, and the electron‐lucent structures, that is, the lipid bodies appear black measuring approximately 0.2–0.3 μm in diameter.

#### 3.1.2 Chemical Imaging by Nano‐SIMS

**FIGURE 2:**
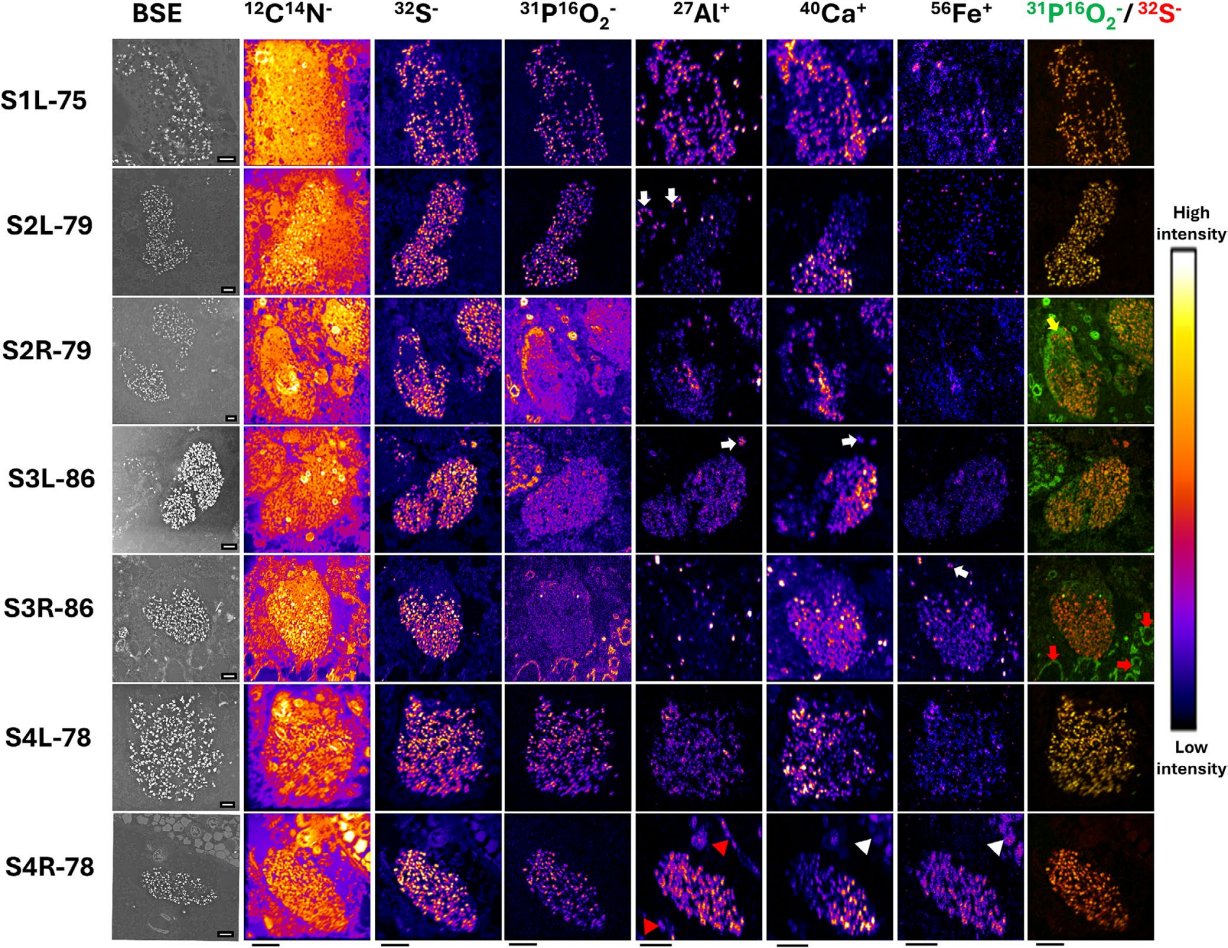
Exemplary BSE images and corresponding qualitative nano‐SIMS maps from each one of the NM‐containing neurons in each of the 7 samples. The distribution of ^12^C^14^N^−^, ^32^S^−^, ^31^P^16^O_2_^−^, ^27^Al^+^, ^40^Ca^+^, and ^56^Fe^+^ isotopic peaks are depicted, with ^31^P^16^O_2_^−^/^32^S‐false‐color overlay of NM‐containing neurons. Field of view (FOV) for negative ion maps: 45 × 45 μm^2^; for positive ion maps: 40 × 40 μm^2^. The images were 2 × 2 binned to a pixel size of 128 × 128 pixels in order to increase signal intensity. Images are displayed in linear intensity scale. Scale bar: 5 μm for BSE images, 10 μm for ion maps. Red arrows: myelin sheaths; yellow arrow: neuron nucleus; white arrows: Extra‐neuronal structures also displaying ^27^Al^+^, ^40^Ca^+^ and ^56^Fe^+^ signal. The arrowheads in the blood vessels of S4R‐78 point to erythrocytes (white arrowhead) and endothelium (red arrowhead). Labels on the left represent the sample ID. Color scale bar represents the signal intensity of the elements, ranging from low (cold color) to high (warm color). The same result was observed in *n* = 3 neurons per sample (not shown).

Elemental analyses of NM‐containing neurons in the 7 samples demonstrated a robust fingerprint pattern in which the intra‐neuronal NM is highly enriched in sulfur (detected as ^32^S^−^ ion), phosphorus (detected as ^31^P^16^O_2_^−^ cluster ion), aluminum (^27^Al^+^), calcium (^40^Ca^+^) and iron (^56^Fe^+^) (Figure 2). In contrast, no relevant signals of copper (^63^Cu^+^) and zinc (^66^Zn^+^) were detected, except for sample S3R‐86 showing a very low copper intensity (Figure S1). NM‐containing organelles also contain melanin‐protein conjugates, as reflected by the carbon nitrogen cluster ion (^12^C^14^N^−^), but not exclusively colocalized with the NM pigment. High ^12^C^14^N^−^ signal is also present within any protein or nucleic acid compartments of biological tissues. Thus, sulfur was considered to be the most appropriate marker to identify NM pigment as outlined in the previous paragraph. The ^31^P^16^O_2_^−^ maps show a direct correlation of phosphorus signal with the sulfur rich regions, as also shown by the direct overlay between the two maps. Phosphorus is also localized in other regions of the LC tissue such as the myelin sheaths and the cell nucleus (Figure 2). Moreover, ^27^Al^+^, ^40^Ca^+^, and ^56^Fe^+^ were also detected in regions outside NM‐containing neurons. In S4R‐78, erythrocytes are also included in the field of view displaying ^56^Fe^+^ and ^40^Ca^+^ signals and the vascular endothelium with high ^27^Al^+^signal (Figure 2).

#### 3.1.3 Semi‐Quantitative Analysis Between Different NM Compartments and Subjects

**FIGURE 3:**
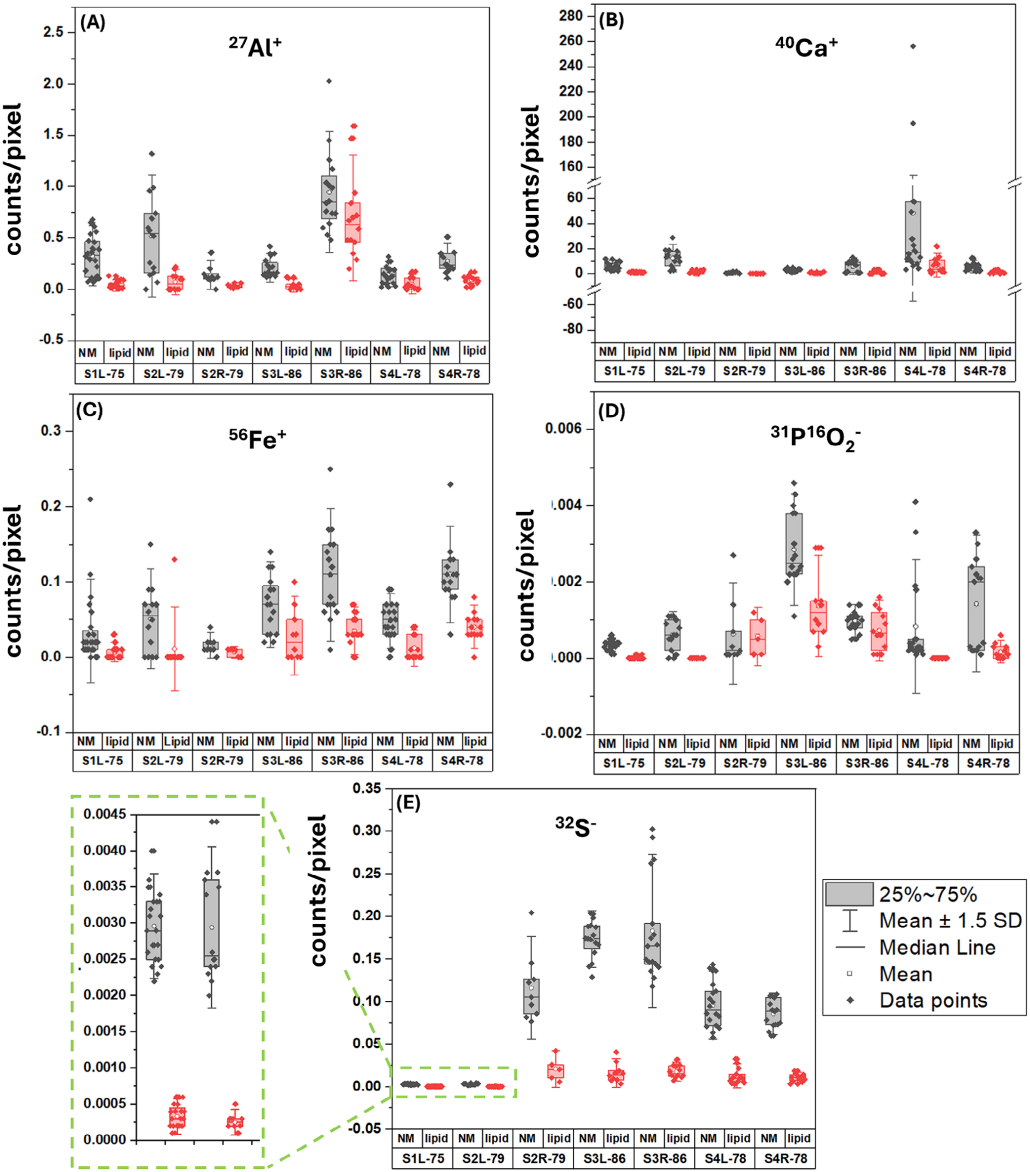
Box plots of the counts per pixel values of (A) ^27^Al^+^, (B) ^40^Ca^+^, (C) ^56^Fe^+^, (D) ^31^P^16^O_2_^−^, and (E) ^32^S^−^ in NM pigment (labeled “NM”, *n* ≥ 5) and lipid bodies (*n* ≥ 5) of the (NM‐containing organelles) across the seven samples. Data were collected from *n* = 3 distinct NM‐containing neurons in each sample. The box plots for NM and lipid groups are color coded with gray and red, respectively.

Based on the spatial distribution of elemental maps—primarily the ^32^S^−^ signal within the NM‐containing neurons—four distinct compartments were identified: NM pigment, lipid bodies, cytoplasm, and embedding resin background signal (see Figure 1B) that were later selected for semi‐quantitative analysis. At least five ROIs per compartment were selected, and counts/pixel ratios were extracted from each ROI, providing element‐specific measurements independent of particle size (Biesemeier et al. 2016). The mean values of the normalized counts per pixel ratios were compared across the compartments as well as across the subjects, and among the two different hemispheres within the same subject (Figure 3).

Higher signals were obtained for all selected elements in NM pigment as compared to lipid compartment. Elemental signals were virtually absent in the cytoplasm and resin, potentially attributable to the washout of water‐soluble molecules during embedding process, except for nitrogen (as ^12^C^14^N^−^), which can be a protein component and is therefore abundant in many biological structures. Among the subjects, the highest accumulation of iron was noticed in NM of subject 3 (86 years old) followed by subject 4 (78 years old). Lowest accumulation was observed in subject 1 (75 years old) and subject 2 (79 years old). For both subjects 3 and 4, the iron and aluminum signals relative to NM were higher in the right hemisphere compared to the left hemisphere, in contrast to subject 2. A trend for a positive correlation between iron and sulfur was observed, however not reaching the level of significance (Spearman (*ρ*) correlation coefficient: 0.35; *p*‐value = 0.45).

### 3.2 Analysis of NM‐Containing Organelles by STEM‐EDX

#### 3.2.1 Chemical Imaging Using STEM‐EDX

**FIGURE 4:**
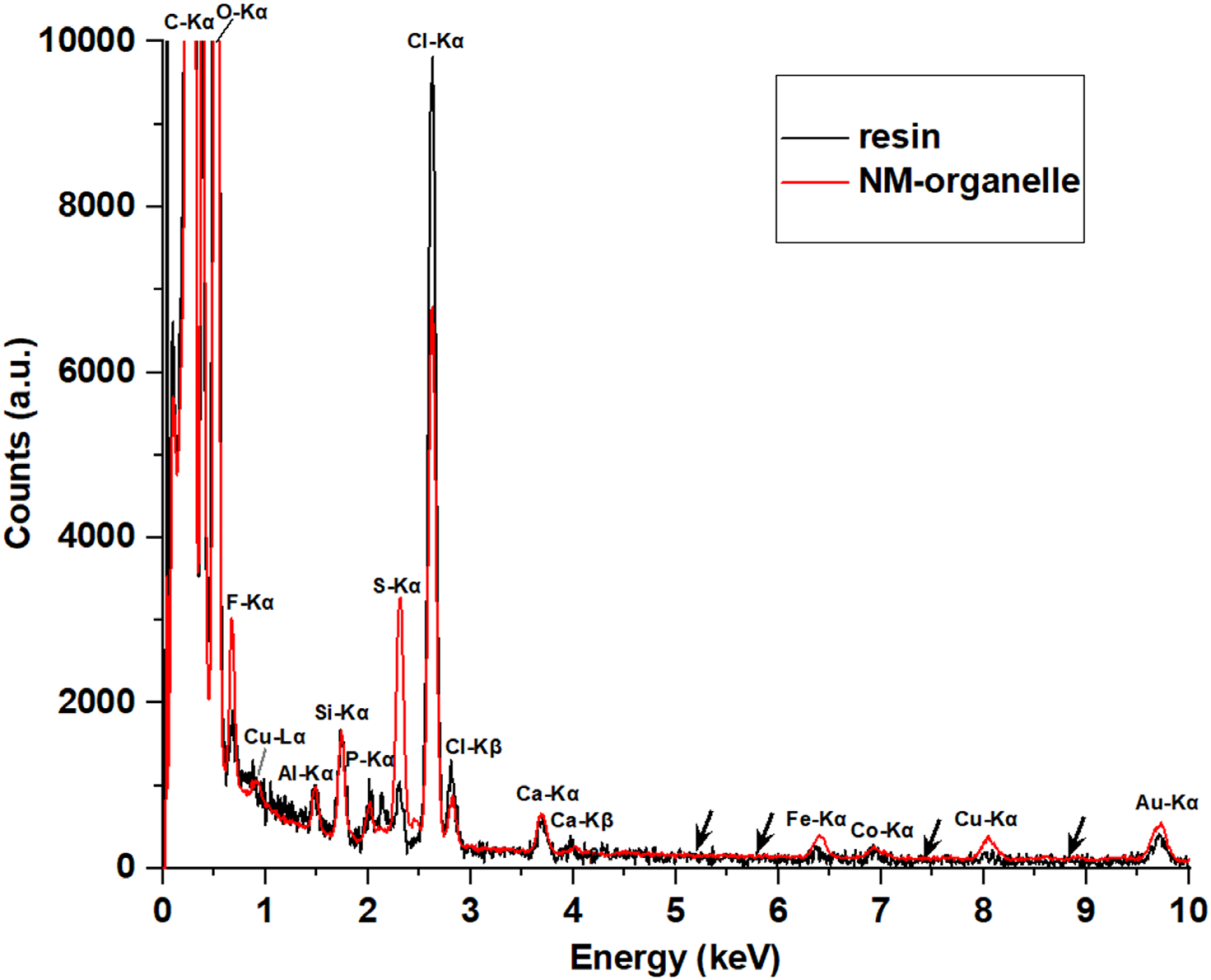
Typical STEM‐EDX spectrum of a ROI containing only pure resin (black) and NM‐containing organelle of an exemplary LC pigmented neuron (red). Artifact peaks are C, O, Si, Al, P from resin embedding, Au from the grid and minor Fe, and Co contributions derive from the TEM column. C, O, N, S, and P are typical structural elements present in any biological material. The NM spectrum presents with higher peaks for S, Fe and other metals (sample dependent) as compared to resin background or cytoplasm (not shown). Black arrows represent an example of the background noise (a peak is significant if > 3× noise). Spectra were adjusted to 10 000 counts max, for this reason some peaks (C, O) look cut and incomplete.

**FIGURE 5:**
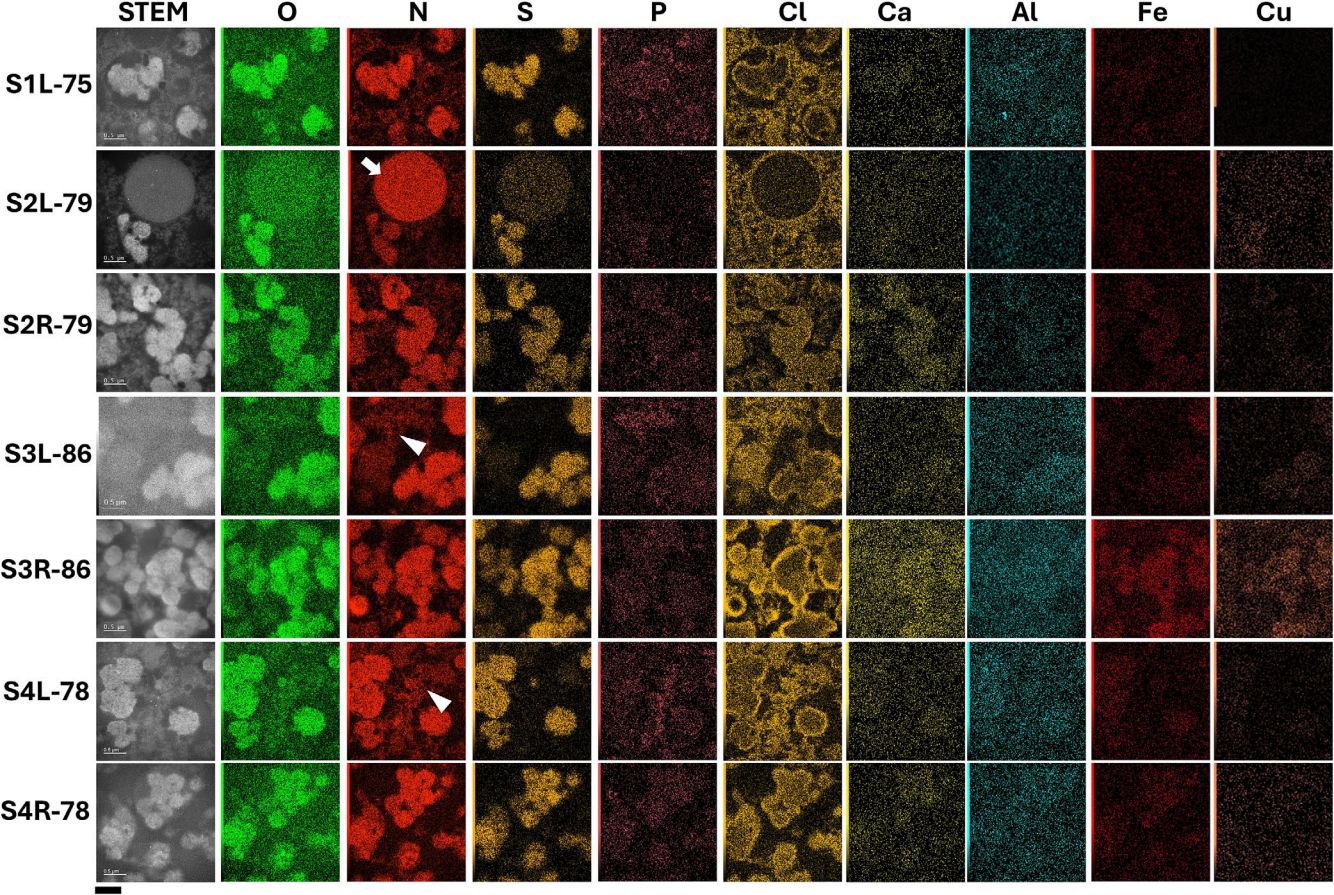
Exemplary STEM‐EDX maps of the O, N, S, P, Cl, Ca, Al, Fe, and Cu K‐shell peaks in NM‐containing organelles of the seven samples, and the corresponding STEM images for the identification of the selected ROI. S, O, and N are primarily localized within the NM pigment and, to a lesser extent, within the nitrogen‐rich protein matrix. Ca, Al, P, Fe, and Cu signals are very weak, but mainly appear associated with the NM pigment, except for P, which is also significantly confined to the nitrogen‐rich protein matrix. In the STEM image, high electron density regions like NM pigment appear white, while regions of low electron density appear black. White arrow: N‐rich noradrenergic vesicles; white arrowheads: protein matrix of NM‐containing organelles. Scale bar = 0.5 μm.

EDX analysis performed in STEM mode allows for even higher resolved mapping of the elemental composition of the sub‐NM compartments based on the emitted X‐rays after electrons impacting the sample. Figure 4 shows a representative integrated STEM‐EDX spectrum of a ROI focusing on a NM‐containing organelle (red) and pure resin background (black). The resin mainly contains carbon, oxygen, and silicon elemental peaks with lower amounts of chlorine and artifact peaks from the gold grid and microscope components (iron, cobalt). Aluminum, phosphorus, and fluorine peaks are also present, which might be due to resin additives or lab tools contamination during resin embedding. Above this background, NM‐containing organelles show prominent sulfur peaks and higher iron and copper peaks. However, a clear enrichment of Al signal in NM as shown by nano‐SIMS was not observed. Elemental maps (Figure 5) revealed that all samples exhibit a strong oxygen signal that was higher in the NM pigment and protein compartments than in the lipid compartment of NM‐containing organelle or the background. This finding is consistent with the fact that main components of lipid bodies are dolichols, which have a lower oxygen content compared to NM and proteins. Nitrogen was also significantly present in the NM pigment (arising from catechol‐derived moieties and melanin‐protein conjugates forming NM), as well as in the protein matrix of NM‐containing organelles (white arrowhead, Figure 5); it was also detected within vesicles with a perfectly round shape (as for S2L‐79, white arrow in Figure 5), which likely represent the noradrenergic vesicles previously observed (Issidorides et al. 2004). Sulfur was mainly confined to the NM pigment of the NM‐containing organelles, with a weaker signal observed also in some protein matrices and in the noradrenergic vesicles. Phosphorus showed a very weak signal in the maps, sometimes colocalizing with that of sulfur, although not specifically related to it. Chlorine signals were mainly found in sulfur and nitrogen‐rich structures, and an interesting pattern of higher chlorine signal was also detected at the edge of NM pigments of the NM‐containing organelles. Calcium, aluminum, and copper also showed a pattern confined to NM pigment, but with a very weak signal in the maps.

#### 3.2.2 Quantitative Analysis of Specific NM‐Containing Areas

**FIGURE 6:**
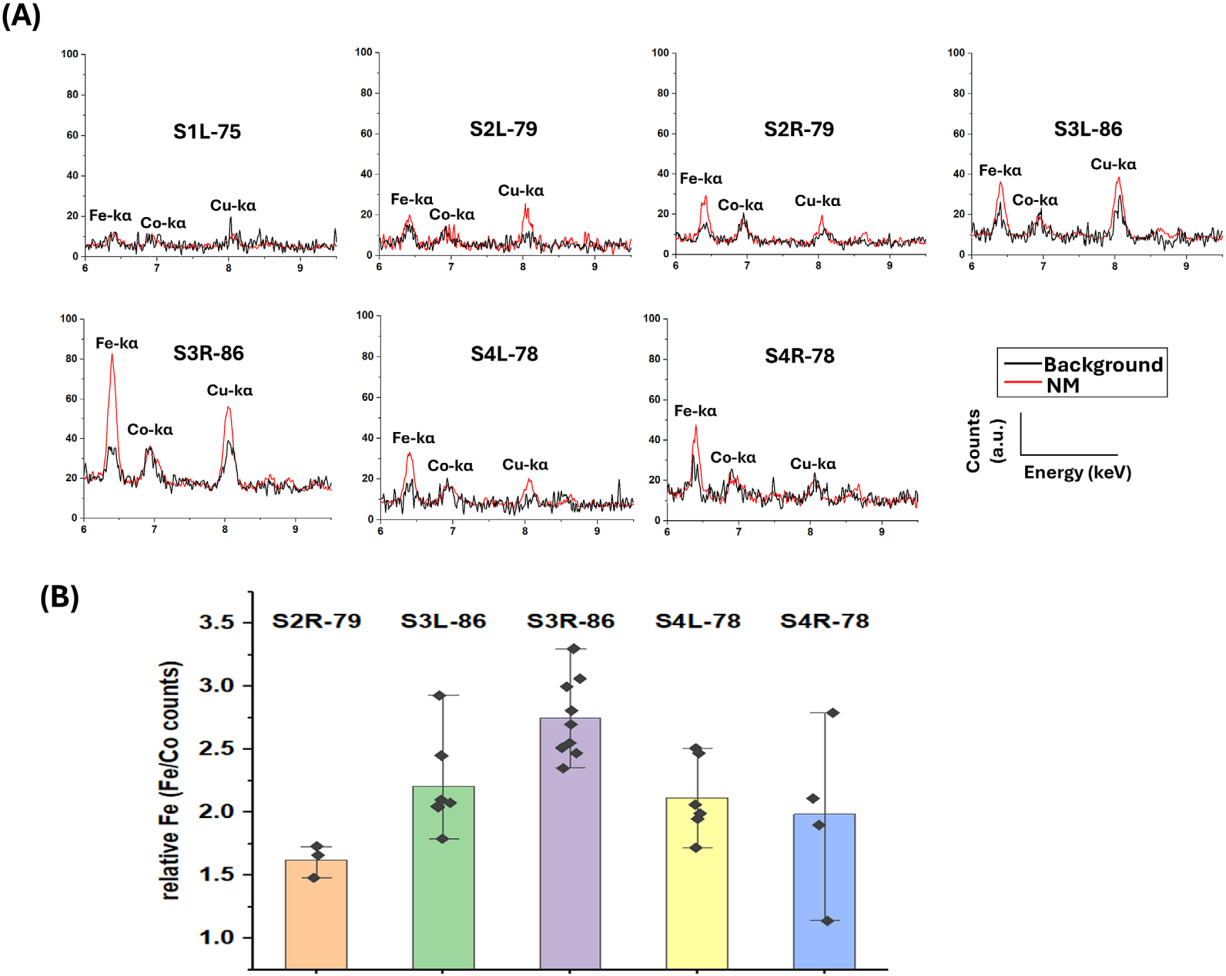
Semi‐quantitative analysis of iron signals across all samples. (A) Mean EDX spectra of NM pigment (NM‐red, *n* ≥ 3) and cytoplasmic background (cytoplasm‐black, *n* ≥ 3) for each sample. The Fe peaks higher in NM than in the corresponding background indicate a true Fe signal. (B) Comparison of relative Fe levels in NM across the 5 samples with relevant iron peaks, in *n* ≥ 3 NM pigments. Fe net counts were normalized to Co counts to account for variability. Error bars correspond to the standard deviation.

While nano‐SIMS provides exclusively qualitative isotopic data sets, which need to be treated to achieve at least semi‐quantitative results, (S)TEM‐EDX per se is a quantitative elemental analysis tool but typically with lower sensitivity, especially for light elements. NM pigment, lipid bodies, and cytoplasmic background corresponding to the void areas in the cells were selected in each map for quantitative analysis and a mean value and standard deviation were calculated (Table 2). While this approach worked well for the NM pigment, signals from lipid bodies were usually very low for all elements. Therefore, quantitative analysis was not performed there. Within the NM pigment, Al, Fe, and Cu had a low atomic percentage in comparison to the structural elements and were close to the EDX detection limit (> 0.02 at% for Fe). As a result, comparing low‐abundance elements (such as iron) between samples and subjects was not straightforward due to limited statistical power. Therefore, another semi‐quantitative approach was implemented to identify relevant amounts of iron and to compare them between samples. For this purpose, an artifact peak was selected (cobalt in this case, since not a component of the specimen), and the spectra from both NM pigment and cytoplasm background from the same sample were normalized to it (Figure 6A). As expected, due to lack of relevant iron peaks in the NM pigment spectra, samples S1L‐75 and S2L‐79 showed exactly the same height of iron peaks between NM pigment and the cytoplasmic background, while relevant peaks were observed for the remaining samples. Iron‐binding by NM varies across individual NM pigments (Figure 6B). Therefore, more than one NM‐pigment deposit was analyzed per sample; and still in S1L‐75 and S2L‐79 measured NM pigments, all the spectra lacked an iron peak. As noted above, a lack of detectable iron signals in EDX does not mean that iron is completely absent from NM; it only indicates that its concentration is below the detection limit of the technique (as it was visible in SIMS). Afterwards, to compare between the samples that had relevant iron peaks, relative Fe levels in NM were calculated (Figure 6B). This revealed a clear trend of iron difference among samples, similar to what was obtained from the nano‐SIMS data: samples with higher nano‐SIMS iron signals generally showed higher iron levels in EDX, and vice versa. The S/Fe correlation was not confirmed from EDX data, likely due to the limited number of measurements, and because the quantitative atomic percentage results for iron are not fully reliable. Copper was observed at low intensity in a few samples, while remaining below detection limit in others. As an estimation for the eumelanin/pheomelanin ratio in the pigment (as explained later in the discussion), the sulfur/nitrogen (S/N) ratio was calculated, and it ranged between 0.14 and 0.19 at% among the different samples.

**TABLE 2:** Quantitative analysis of EDX mappings in at% for the structural elements and metals contained in the NM pigment of the NM‐containing organelles in LC neurons. For each single sample, *n* ≥ 3 selected NM pigment areas were considered. The values marked by asterisks correspond to the elements with atomic percentages at/below the detection limit. Data are presented as mean ± standard deviation.

	S1L‐75	S2L‐79	S2R‐79	S3L‐86	S3R‐86	S4L‐78	S4R‐78
C‐Kα	87.9 ± 2.55	88.9 ± 1.03	87.9 ± 3.18	88.7 ± 1.40	90.4 ± 0.54	91.1 ± 0.89	89.4 ± 1.30
N‐Kα	6.03 ± 0.62	5.57 ± 0.46	6.28 ± 0.65	5.76 ± 0.78	4.61 ± 0.25	4.56 ± 0.32	5.50 ± 0.76
O‐Kα	4.88 ± 2.06	4.28 ± 0.43	4.13 ± 3.40	4.15 ± 0.45	3.67 ± 0.25	3.13 ± 0.45	3.81 ± 0.37
Al‐Kα	0.02 ± 0.00*	0.02 ± 0.01*	0.00 ± 0.00*	0.02 ± 0.00*	0.02 ± 0.01*	0.03 ± 0.00*	0.02 ± 0.00*
P‐Kα	0.00 ± 0.01*	0.04 ± 0.02*	0.06 ± 0.01*	0.03 ± 0.00*	0.03 ± 0.00*	0.03 ± 0.00*	0.04 ± 0.01*
S‐Kα	0.93 ± 0.19	0.79 ± 0.19	0.96 ± 0.18	1.03 ± 0.23	0.64 ± 0.12	0.87 ± 0.08	0.83 ± 0.12
Cl‐Kα	0.22 ± 0.07	0.20 ± 0.03	0.31 ± 0.07	0.23 ± 0.04	0.44 ± 0.6	0.22 ± 0.03	0.21 ± 0.03
Ca‐Kα	0.06 ± 0.01	0.13 ± 0.01	0.25 ± 0.07	0.06 ± 0.01	0.12 ± 0.01	0.07 ± 0.01	0.12 ± 0.01
Fe‐Kα	0.00 ± 0.00*	0.00 ± 0.00*	0.02 ± 0.00*	0.02 ± 0.00*	0.03 ± 0.01*	0.02 ± 0.00*	0.02 ± 0.00*
Cu‐Kα	0.00 ± 0.01*	0.03 ± 0.00*	0.02 ± 0.01*	0.03 ± 0.01*	0.03 ± 0.00*	0.01 ± 0.00*	0.01 ± 0.01*
S/N	0.15 ± 0.00	0.14 ± 0.00	0.15 ± 0.00	0.18 ± 0.00	0.14 ± 0.00	0.19 ± 0.00	0.15 ± 0.00

## 4 Discussion

### 4.1 High‐Resolution Elemental Imaging: A Valuable Tool in Neurochemistry

Although molecular investigations on NM are crucial for understanding brain aging and age‐related neurodegenerative diseases, most studies in this context were performed on NM isolated from the SN. In LC, data on NM is very limited due to difficulties in obtaining sufficient material and reliance on pooled samples that obscure individual and compartment‐specific differences (Zecca et al. 2004). Moreover, isolation steps can also cause elemental redistribution that interfere with analysis. Therefore, spatially resolved analysis of NM in intact tissue, as performed here, is essential to provide elemental information at the sub‐organellar level while avoiding artifacts introduced by NM extraction procedures. As discussed in the introduction, several studies claimed high spatial resolution chemical imaging for SN‐NM, but most could not reach below 500 nm of detail. To our knowledge, only one study has achieved elemental mapping of SN‐NM at a 100–150 nm lateral resolution using a correlative approach integrating nano‐SIMS mapping with EDX/EELS point spectra (no EDX maps, as shown in Biesemeier et al. 2016). Hence, a similar but more advanced approach was applied here to study LC‐NM for the first time, providing information on the elemental backbone composition of NM‐containing organelles and their sub‐compartments in intact LC tissue, as well as their metal‐binding capacity, at lateral resolutions of 150–200 nm for nano‐SIMS and ~5–10 nm for EDX mapping. For direct comparison, one SN sample (84 years old female) prior investigated (Biesemeier et al. 2016) was reinvestigated here. Respective spectra and elemental maps are presented in Figure S2. The high‐resolution capability was evident in the results of this study, since we were able to clearly discriminate between the lipid bodies, protein matrix and NM pigment of the NM‐containing organelles and gained insights into different individual cells and brain hemispheres of the same LC samples. The quantitative (for EDX) and semi‐quantitative (for nano‐SIMS) data obtained here are also of great importance to compare elemental distributions among the samples from different elderly subjects. Future studies with larger cohorts of samples will reach a better understanding on how NM metal accumulation is affected in aging and with disease.

### 4.2 Nano‐SIMS Plus EDX: Synergistic Analytical Approaches for Enhanced Characterization of Low Abundance Elemental Signatures

In this study, a proof‐of‐concept correlative approach was employed for the elemental analysis of NM‐containing organelles within the LC, with the combination of optical and electron microscopy, EDX and SIMS mapping performed on epoxy‐embedded cross sections. Contrast enhancement like heavy metal stains typically employed for tissue cross sections (e.g., BSE‐SIMS by He et al. 2017) was not used to avoid interference coming from introduced metal signals in the analysis. The naturally auto‐contrasting NM could directly be visualized by optical microscopy, and ROIs investigated by downstream analytical methods on the same sections. For STEM‐EDX, NM also inherently provided sufficient contrast for STEM imaging modes, and even other cellular compartments were sufficiently visible with low‐kV STEM without staining. Only for ultrastructural imaging using BSE, post nano‐SIMS staining was employed on consecutive sections, as nano‐SIMS is destructive and can ablate the ROI.

Combining multiple chemical imaging techniques proved also a more effective approach for more significant and reliable data, mainly when comparing the results among the different subjects: while nano‐SIMS was more sensitive than EDX for most of the elements, EDX provides higher spatial resolution and direct quantification of spectral information. The absence of a peak for a low‐abundance element in a given technique does not necessarily exclude its presence in the sample; rather, it might be traced by a more sensitive approach. Thus, due to their small size and limited signal intensities from those ROIs, quantification was not possible for lipid bodies with EDX, but possible with nano‐SIMS using a semi‐quantitative approach. In the case of some metal (e.g., Fe) distributions and quantifications, the semi‐quantitative SIMS data, however, could be confirmed with EDX assuring the suggested fingerprint pattern among the samples.

Despite the methodological advantages, potential redistribution of ions during chemical fixation and resin embedding procedures always needs to be considered in chemical imaging studies. However, the presented analyses focused on NM‐containing organelles, in which metals are believed to be strongly chelated within the melanin (Hong and Simon 2007) and NM complexes (Double et al. 2003; Zecca, Bellei, et al. 2008; Zecca et al. 1996). Previous EDX studies comparing conventional glutaraldehyde‐fixed/resin‐embedded and cryo‐preserved ocular melanin samples reported only ~10%–20% differences for Zn, Fe, Ca, and Cu between the two procedures, suggesting that melanin‐bound metals are not significantly altered by conventional preparation procedures (Samuelson et al. 1993). However, some degree of fixation‐related alteration cannot be entirely excluded.

### 4.3 Identification of Structural Components of NM‐Containing Organelles by Chemical Imaging

Elemental analysis of the LC tissues from the seven samples demonstrated that NM‐containing organelles are rich in sulfur, mainly coming from the benzothiazine and benzothiazole blocks of pheomelanin moieties in the NM pigment (Wakamatsu et al. 2003, 2012; Zecca et al. 1992). As highlighted by both nano‐SIMS and EDX, sulfur is localized within the NM pigments but also, in part, in the protein portion constituting both the melanin‐protein adducts within the NM structure and the protein matrix of the organelle (much clearer in EDX). This agrees with previous data of sulfur content contributed by benzothiazine/benzothiazole moieties and by cysteine/cystine units of protein components of NM (Zecca et al. 2000). In the past, a sulfur to nitrogen (S/N) ratio of 0.02 or lower was considered for pure eumelanin as studied in hair and melanoma analyzed by liquid chromatography (Ito and Fujita 1985) and sepia melanin analyzed by combined EDX and Electron Energy Loss Spectroscopy (Biesemeier et al. 2011). A S/N ratio of 0.4 was obtained for pure pheomelanin studied in melanosomes of malignant melanoma (Jimbow et al. 1984). Therefore, an increasing S/N ratio represents an increasing pheomelanin fraction in the NM pigment. Here, the S/N ratios ranged from 0.14 to 0.19, which is (1) close to the calculated value of 0.2 in the theoretical structural formula of NM of SN (SN‐NM: C48.1H70.2N5.72O16.8S (mMW = 1029.4), with H/C molar ratio = 1.46) (Engelen et al. 2012). (2) However, it is lower than the measured S/N ratio reported in the same study for isolated SN‐NM analyzed using combustion techniques (S/*N* = 0.4) (Engelen et al. 2012). (3) It is slightly higher than the S/N ratio detected for SN‐NM using TEM EDX‐EELS at 120 kV (0.11; Biesemeier et al. 2016). As shown by chemical degradation, the melanic component of NM is mixed pheomelanin/eumelanin with a ratio 1/3 (Wakamatsu et al. 2003). However, in addition to pheomelanin itself, NM contains eumelanin and a protein component which further decreases the S content with respect to pure pheomelanin component (Zecca et al. 2000; Wakamatsu et al. 2015).

Phosphorus, detected as the ^31^P^16^O_2_^−^ cluster ion in nano‐SIMS, was largely restricted to the NM pigment, with minimal signals in the lipid compartments of the NM‐containing organelles of LC. This contrasts with previous nano‐SIMS studies in SN neurons, where phosphorus localized within NM pigment, but was more pronounced in the lipid bodies of the NM‐containing organelles (Biesemeier et al. 2016). EDX mapping of LC in the present study and the reinvestigation of one SN tissue of our previous study (see Figure S2) confirmed strongest P signals in pigment and protein‐rich structures of the NM‐containing organelles rather than lipid bodies for both brain regions. This suggests that NM of both regions can sequester phosphorus‐containing molecules, likely from phospholipid‐related pathways involved in the organelle formation (Zucca et al. 2018). The low phosphorus signal in LC lipid compartments of NM organelles likely reflects concentrations below detection limits and/or indicates possible differences in phospholipid distribution compared with those of SN, whose lipid fractions contained some phospholipids (Zucca et al. 2018), although P is clearly present in phospholipid‐rich structures such as myelin and nuclei. Studies on the NM pigments isolated from putamen, globus pallidus, premotor cortex, cerebellum, SN, and LC using nuclear magnetic resonance and liquid chromatography‐mass spectrometry, have shown that among the lipids adsorbed to NM pigments, the dolichols and dolichoic acids represent the major portion, with highest content in the SN and lowest in the LC (Engelen et al. 2012; Ward et al. 2009; Zecca, Bellei, et al. 2008). A higher abundance of dolichols and dolichoic acids compared to other lipid classes, including phospholipids, has also been confirmed using liquid chromatography‐mass spectrometry and thin‐layer chromatography in the lipid portion of NM pigment and NM‐containing organelles isolated from SN neurons (Zucca et al. 2018). Another study, from the same group, using infrared spectroscopy suggested that NM of the SN and that of the LC seem to contain less gangliosides (lactones) and phospholipids than NMs isolated from other pigmented brain regions like putamen, premotor cortex, globus pallidus, cerebellum, etc. (Engelen et al. 2012). Taken together, these data suggest that the concentration of phospholipids in the lipid compartment of NM‐containing organelles in the LC is low and likely falls below the detection limits of both instruments used here.

Chlorine was detected with a notable localization pattern in the EDX maps. Despite its presence in NM, chlorine signal was higher on the edges of NM pigments, like a marked boundary surrounding the pigment itself. This pattern of chlorine signal was not observed in the protein part (the N‐rich part) of the NM, where Cl signal appeared homogeneous. Therefore, this might explain a preferential ionic interaction of chloride to NM at different exposed sites. The accumulation of chloride ion, coming from cytosol, at the border of NM pigments is due to repulsion by low polarity NM and lipid bodies contained inside the organelle, while chloride is homogeneously distributed over N positive charged groups of proteins. In aging there is an increase of highly charged proteins that are at risk for oxidative damage (de Graff et al. 2016).

### 4.4 Metal Sequestration by NM: Interindividual Differences

NM pigments from neurons of different brain regions are known to chelate metal ions, like calcium, aluminum, iron, copper, zinc, and others (Zecca, Bellei, et al. 2008; Zecca et al. 1994, 1996; Zecca and Swartz 1993). In this study, Ca, followed by Fe and Al, were the most common metals detected in all samples. Among the subjects, no specific aging trend was observed for Ca and Al; in contrast, a potential specific age‐related difference was observed for Fe, with the highest level of iron accumulation in the elder subject (S3‐86), followed by subject S4‐78, and the lowest observed in the 75 and 79 years old subjects (S1‐75 and S2‐79), respectively. Although based on a very small sample size and limited statistical significance, this analysis provides preliminary evidence that (1) iron is significantly accumulating only in the pigment part of the NM organelle in intact LC neurons, (2) the technique is able to differentiate both intra‐ and interpersonal changes regarding gross iron levels and (3) would allow age‐related assessments in a larger pool of subjects. A previous study, including several subjects with a wide age range, has shown that total iron content (including both NM‐bound iron and that accumulated in iron‐containing proteins) in LC brain area remains stable during aging averaging approximately 29 ng/mg of wet LC tissue (Zecca et al. 2004).

It is well known that in PD, the levels of potentially toxic metals, mainly iron, increase in the SN (Dexter et al. 1989; Riederer et al. 1989; Faucheux et al. 2003; Gaurav et al. 2025; Guan et al. 2022; Zucca et al. 2026) and the pro‐oxidant pheomelanin portion of the NM pigment also increases in the SN of PD patients compared to healthy controls (Cai et al. 2023). Assessment on whether healthy aged subjects showed a similar pattern (i.e., whether samples with higher iron levels had also increased sulfur signals derived from pheomelanin portion of NM) showed a positive, however non‐significant trend (Fe^+^/S^−^, *ρ* =0.35). Direct linear Fe‐S regression could not be performed for EDX results due to low Fe signals.

Regarding the less abundant metals found in LC‐NM in this study, Cu always fell below the nano‐SIMS detection limit, except for sample S3R‐86 that showed a very weak signal (Figure S1). Also, in EDX, very weak Cu peaks were detected in maps and spectra (as well as in the SN tissue re‐investigated for comparison by STEM‐EDX mapping in Figures S2 and S3). This contrasts with the prior SIMS‐EDX analyses, where Cu was clearly present in SIMS maps and EDX spectra obtained with a spot size of 100 nm within the NM pigment (Biesemeier et al. 2016). The concentration of copper in isolated LC‐NM was found to be higher than that measured in isolated SN‐NM (Zecca et al. 2004). However, a lower amount of Cu compared to Fe was also found in NM isolated from both the SN and LC (Zecca et al. 2004), and this observation was explained as the consequence of reducing conditions in postmortem brain tissues which would reduce Cu (II) to Cu (I) that has lower affinity for NM (Zecca et al. 1996). A very weak copper signal in the NM of SN, which was lower in a PD sample compared to a control case, was also reported by Reinert and colleagues, who proposed that this copper reduction in NM could display lower content of copper in the intraneuronal environment, which is linked to reduced copper/zinc‐superoxide dismutase enzymatic activity, finally leading to increased oxidative stress (Reinert et al. 2007). Moreover, zinc was not detected in the present study with either method.

### 4.5 Metal Sequestration by NM: Intra‐Individual Differences

When comparing data sets between the two different LC hemispheres in the same individuals, a trend in the metal accumulation and the sulfur‐related composition in pigmented neurons was observed. For two subjects, Fe and Al were higher in the right LC hemisphere in comparison to the left one (not for S2‐79, which had fewer data points, see Figure 3). Also, the relatively potent quantitative EDX data for calcium showed approximately doubled calcium atomic percentages in the right hemisphere of all subjects as compared to the left one, suggesting asymmetry in elemental distributions within NM‐containing organelles between left and right sides of healthy LC. This might be one of the reasons why the age‐related neurodegenerative processes start in one hemisphere in the first place, leading to symptoms in one body side in the early stages of PD (Djaldetti et al. 2006). One investigation discovered that atrophy in the cortical region starts in the left frontal regions in early stages of PD (Claassen et al. 2016). NM‐sensitive MRI, which is able to detect NM‐iron complexes due to their paramagnetic properties (Sasaki et al. 2006; Sulzer et al. 2018; Trujillo et al. 2017; Zecca et al. 1996) showed different contrast on left and right SN sides, corresponding to a differential neuronal degeneration, and this was linked to the asymmetry in handedness and motor symptoms of PD (Shinde et al. 2019). Similar NM‐MRI results are reported also for the LC, showing differences between left and right sides (Liu et al. 2023).

## 5 Conclusion

This study employed a correlative workflow using dark‐field light microscopy and high‐resolution electron microscopy, nano‐SIMS, and EDX to analyze the composition of NM‐containing organelles within seven LC tissue sections from different aged control subjects. Chemical imaging data revealed that elements like C, N, O, P, S, Cl, Fe, Ca, Al, and Cu localized to NM‐containing organelles, with lateral resolutions allowing distinction between their lipid, protein, and NM pigment compartments. Our analyses showed that the S‐rich pigment compartment is primarily responsible for accumulating metals (like Fe, Ca, Al) as well as P, that could be indicative of any phosphorous‐containing molecules, for example phospholipids.

Moreover, a methodology for quantitative EDX and semi‐quantitative nano‐SIMS data analysis was applied, demonstrating inter and intrapersonal differences of metals, with a positive trend for iron and sulfur (pheomelanin) to age. Both techniques produced consistent results, and the combination of approaches strengthened the robustness of findings, despite a limited number of samples and low signal intensities for low abundance elements.

This study establishes a foundational workflow that can support future investigations of NM in the LC using larger cohorts. It also provides a framework for examining intraneuronal elemental distributions in PD tissue, without relying on isolated NM, therefore allowing comparison across individual NM organelles, affected neurons, hemispheres, age groups, and disease stages.

The current study paves the way for future analysis of LC tissues of PD patients to assess whether there is also a variation in the elemental composition among pathological and control subjects, and thus to understand the role of metal accumulation and chelation in the susceptibility of this brain region in disease progression. For future perspectives, mass spectrometry imaging methods could be used to provide metal and non‐metal analysis for other neurodegenerative diseases like AD, multiple sclerosis, progressive supranuclear palsy, and acute macular degeneration, providing valuable insights in disease mechanisms and potentially contributing to the development of improved diagnostic and therapeutic strategies.

## Author Contributions

**David Bouvier:** writing – review and editing. **Anaïs Carpentier:** investigation. **Zahraa Berro:** investigation, writing – original draft, methodology, visualization, writing – review and editing, formal analysis. **Dirk Schaumlöffel:** writing – review and editing. **Fabio A. Zucca:** conceptualization, investigation, writing – review and editing. **Antje Biesemeier:** conceptualization, methodology, writing – review and editing, investigation. **Adrian‐Marie Philippe:** investigation, writing – review and editing. **Maria Angels Subirana:** investigation, writing – review and editing. **Michel Mittelbronn:** writing – review and editing. **Andrea Capucciati:** investigation. **Luigi Zecca:** conceptualization, investigation, writing – review and editing. **Jean‐Nicolas Audinot:** writing – review and editing.

## Funding

This work was supported by the National Research Fund Luxembourg through the grant CORE C21/BM/15754743. Nano‐SIMS analyses were supported through the grant INTER/ANR/18/12545362 of the project ANR‐18‐CE34‐0015 of the Agence Nationale de la Recherche, France. Michel Mittelbronn would like to thank the Luxembourg National Research Fond (FNR) for the support (FNR PEARL P16/BM/11192868 grant). This study was also supported by the Pezzoli Foundation for Parkinson's disease (Milan, Italy).

## Conflicts of Interest

The authors declare no conflicts of interest.

## Supporting information


**Figure S1:** contains additional nano‐SIMS maps for ^63^Cu^+^and ^66^Zn^+^and the corresponding BSE image obtained in the same ROI as for the data presented in Figure 2.
**Figure S2: and S3** contain STEM‐EDX maps and the normalized EDX spectra of N, Al, P, S, Cl, Al, Fe, and Cu K alpha‐shell peaks in the NM‐containing organelles of a SN sample (84 year old, female) that was previously investigated as part of (Biesemeier et al. 2016).

## Data Availability

The data that support the findings of this study are available from the corresponding author upon reasonable request.
