# Supplementary material for: High‐Resolution Elemental Analysis of Neuromelanin‐Containing Organelles in Human Locus Coeruleus
Source: J Neurochem. 2026 Jul 27;170(7):e70531. doi: 10.1111/jnc.70531 (PMC13408184; doi:10.1111/jnc.70531)
Supplement: Supplementary file 1 — Figure S1: contains additional nano‐SIMS maps for 63Cu+and 66Zn+and the corresponding BSE image obtained in the same ROI as for the data presented in Figure 2. Figure S2: and S3 contain STEM‐EDX maps and the normalized EDX spectra of N, Al, P, S, Cl, Al, Fe, and Cu K alpha‐shell peaks in the NM‐containing organelles of a SN sample (84 year old, female) that was previously investigated as part of (Biesemeier et al. 2016). [file JNC-170-e70531-s001.docx]

**Supplementary Information**

**High-resolution Elemental Analysis of Neuromelanin Organelles in Human Locus Coeruleus**

**Zahraa Berro^1,2,*^, Fabio A. Zucca^3*^, Maria Angels Subirana^4^, Adrian-Marie Philippe^5^, Dirk Schaumlöffel^4^, Anaïs Carpentier^6^, Andrea Capucciati^7,8^, David Bouvier^6,9^, Michel Mittelbronn^2,6,10,11^, Jean-Nicolas Audinot^1^, Luigi Zecca^3,8^ and Antje Biesemeier^1,§^**

^1^Advanced Instrumentation for Nano-Analytics, Scientific Instrumentation and Process Technology, Luxembourg Institute of Science and Technology (LIST), Belvaux, Luxembourg.

^2^Faculty of Science, Technology and Medicine (FSTM), University of Luxembourg, Belval, Luxembourg

^3^Institute of Biomedical Technologies, National Research Council of Italy, Segrate, Milan, Italy.

^4^Institut des Sciences Analytiques et de Physico-Chimie pour l'Environnement et les Matériaux, (IPREM) UMR 5254, CNRS, Université de Pau et des Pays de l'Adour, Pau, France.

^5^Advanced Characterization of Surface, Interface and Structure, Advanced Analysis and Support, Luxembourg Institute of Science and Technology, Belvaux, Luxembourg

^6^National Center of Pathology, Laboratoire national de santé, Dudelange, Luxembourg.

^7^Department of Chemistry, University of Pavia, Pavia 27100, Italy

^8^Pezzoli Foundation for Parkinson’s disease (Milan, Italy)

^9^Luxembourg Centre of Systems Biomedicine (LCSB), Esch-sur-Alzette, Luxembourg.

^10^Department of of Health, Medicine and Life Sciences (DHML); University of Luxembourg,Esch-sur-Alzette, Luxembourg.

^11^Division of Neuropathology, Department of Pathology and Neuropathology, Medical Faculty, University of Cologne, Germany

*Shared first-authorship

^§^ corresponding author: Advanced Instrumentation for Nano-Analytics, Scientific Instrumentation and Process Technology, Luxembourg Institute of Science and Technology (LIST), 42, rue de Brill, 4422 Belvaux, Luxembourg, antje.biesemeier@list.lu

**Supplemental material contents**

**Supplementary figure 1 contains** additional nano-SIMS maps for ^63^Cu^+^and ^66^Zn^+^and the corresponding BSE image obtained in the same ROI as for the data presented in Fig. 2.

**Supplementary figure 2 and 3 contain** STEM-EDX maps and the normalized EDX spectra of N, Al, P, S, Cl, Al, Fe and Cu K alpha-shell peaks in the NM-containing organelles of a SN sample (84 y.o., female) that was investigated as part of our previous study (Biesemeier *et al.*, 2016).


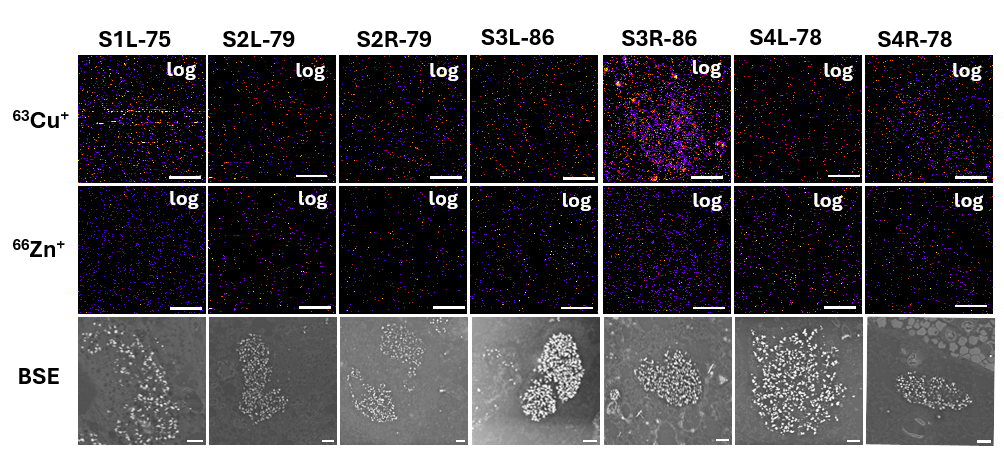


**Supplementary Figure 1.** 40 x 40 μm^2^ field of view nano-SIMS maps for ^63^Cu^+^and ^66^Zn^+^and the corresponding BSE image (as presented in Fig. 2). The maps were 2 x 2 binned from 256 x 256 pixels to a pixel size of 128 x128 pixels. The maps are presented in (log₁₀) scale for contrast enhancement. Scale bar =10 μm for nano-SIMS maps and 5 μm for BSE.


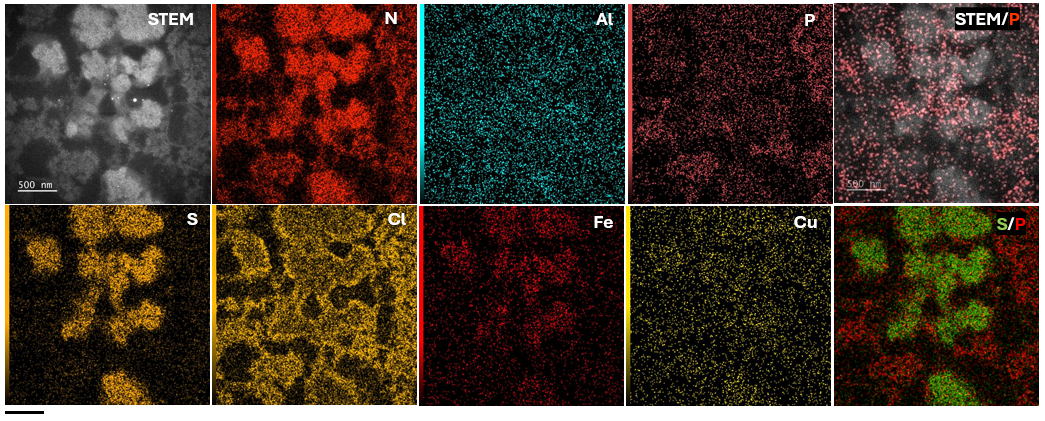


**Supplementary Figure 2.** STEM-EDX maps of the N, Al, P, S, Cl, Al, Fe and Cu K alpha-shell peaks in the NM-containing organelles of a SN sample (84 y.o., female) that was investigated as part of our previous study (Biesemeier *et al.*, 2016), and the corresponding STEM image, and a STEM/P and S/P false color overlay images. Scale bar = 0.5 μm.


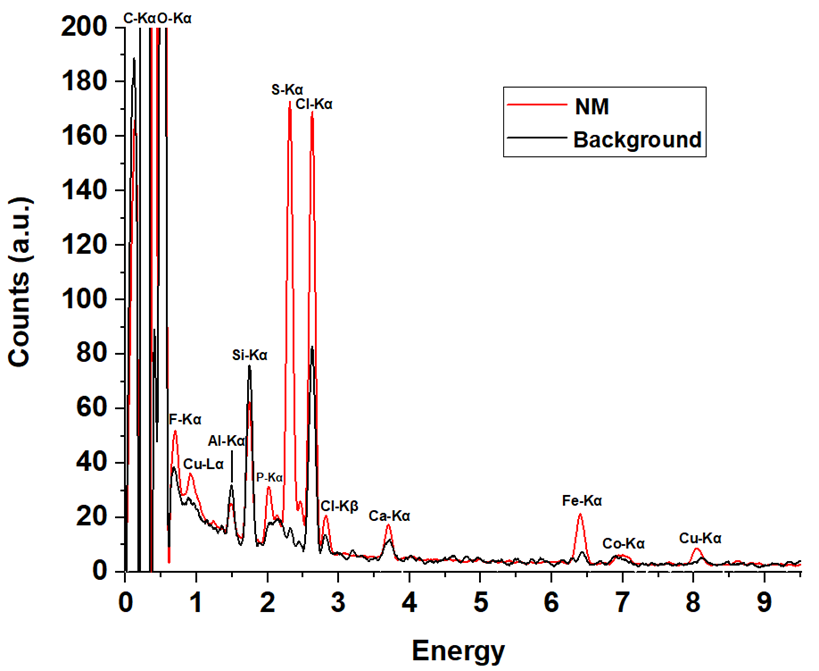


**Supplementary figure 3**. Normalized EDX spectra from SN sample (84 y.o., female) comparing NM pigment (red line) and resin background void areas (black line). A mean spectrum of n ≥ 3 of each area is presented.
